# Supplementary material for: Gochujang Ameliorates Hepatic Inflammation by Improving Dysbiosis of Gut Microbiota in High-Fat Diet-Induced Obese Mice
Source: Microorganisms. 2023 Mar 31;11(4):911. doi: 10.3390/microorganisms11040911 (PMC10141003; doi:10.3390/microorganisms11040911)
Supplement: Supplementary file 1 [file microorganisms-11-00911-s001.zip › microorganisms-2294532-supplementary.pdf]

Table S1. *Gochujang* compositions

	HBM	DBM
Carbohydrate (g/100g)	41.52	39.02
Protein (g/100g)	5.62	5.86
Fat (g/100g)	2.83	3.01
NaCl (g/100g)	6.6	6.8
Calorie (kcal/100g)	214.03	206.61

Table S2. Diet compositions

	ND	HD	SALT	HBM	DBM
Casein (g)	200	200	200	195.7	195.5
L-Cystine (g)	3	3	3	3	3
Corn starch (g)	506.2	0	0	0	0
Maltodextrin 10 (g)	125	125	125	93	94.9
Sucrose (g)	72.8	72.8	72.8	72.8	72.8
Cellulose (g)	50	50	50	50	50
Soybean oil (g)	25	25	25	25	25
Lard (g)	20	245	245	242.8	242.7
Mineral mix (g)	10	10	10	10	10
Vitamin (g)	10	10	10	10	10
Choline bitartrate (g)	2	2	2	2	2
Dicalcium phosphate (g)	13	13	13	13	13
Calcium carbonate (g)	5.5	5.5	5.5	5.5	5.5
Potassium citrate (g)	16.5	16.5	16.5	16.5	16.5
Gochujang (g)				77.00	77.00
NaCl (g)			5.4		
Total (g)	1059.00	777.80	783.23	816.32	817.92

Table S3. Primer list for PCR

Gene Name	Primers	Sequence (5′→3′)
IL-1β	Forward	CACCTTCTTTTCCTTCATCTTTG
	Reverse	GTCGTTGCTTGTCTCTCCTTGTA
TNF-α	Forward	ACTGAACTTCGGGGTGATTG
	Reverse	GCTTGGTGGTTTGCTACGAC
NF-κB	Forward	GAATTCAGTCACTGGCCTCC
	Reverse	TTCAAGACAAAGGAGGTCTGTTT
COX-2	Forward	ACCCCCTGCTGCCCGACACCT
	Reverse	CCAGCAACCCGGCCAGCAATC
β-actin	Forward	AGC CTT CCT TCT TGG GTA TGG
	Reverse	CAC TTG CGG TGC ACG ATG GAG

IL, interleukin; TNF-*α*, tumor necrosis factor alpha; NF-κB, nuclear factor kappa B; COX-2, cyclooxygenase-2.

Supplementary. Biogenic amin of gochujang (mg/kg)

	HBM	DBM
Histamine	160.7	199.09
Tyramine	16.92	22.69
